# Cardiovascular Impairment in COVID-19: Learning From Current Options for Cardiovascular Anti-Inflammatory Therapy

**DOI:** 10.3389/fcvm.2020.00078

**Published:** 2020-04-30

**Authors:** Lun Wang, Yang Zhang, Shuyang Zhang

**Affiliations:** Department of Cardiology, Peking Union Medical College Hospital, Peking Union Medical College & Chinese Academy of Medical Science, Beijing, China

**Keywords:** Coronavirus Disease 2019, cardiovascular impairment, inflammation, cardiovascular diseases, cardiovascular anti-inflammatory therapy

## Abstract

In December 2019, Coronavirus Disease 2019 (COVID-19) caused by SARS-CoV-2, occurred in China and has currently led to a global pandemic. In addition to respiratory involvement, COVID-19 was also associated with significant multiple organ dysfunction syndrome (MODS). Cardiovascular impairment has been observed and is now drawing growing attention. Cardiovascular protective strategies are urgent and of great significance to the overall prognosis of COVID-19 patients. Direct viral infection, cytokine storm, and aggravation of existing cardiovascular diseases were recognized as possible mechanisms of cardiovascular impairment in COVID-19. Hyperactivated inflammation plays an important role in all three mechanisms and is considered to be fundamental in the development of cardiovascular impairment and MODS in COVID-19. Therefore, in addition to conventional cardiovascular treatment, anti-inflammatory therapy is a reasonable strategy for severe cases to further enhance cardiovascular protection and potentially mitigate MODS. We reviewed the inflammatory features and current promising treatments of COVID-19 as well as cardiovascular anti-inflammatory therapies that have been verified in previous clinical trials with positive outcomes. We believe that targeting the central pathway (IL-1β, TNF-α, IL-6), balancing the Th1 and Th2 response, and administering long-term anti-inflammatory therapy might be promising prospects to reduce cardiovascular impairment and even MODS during the acute and recovery phases of COVID-19. The cardiovascular anti-inflammatory therapies might be of great application value to the management of COVID-19 patients and we further propose an algorithm for the selection of anti-inflammatory therapy for COVID-19 patients with or at high risk of cardiovascular impairment. We recommend to take the experiences in cardiovascular anti-inflammatory therapy as references in the management of COVID-19 and conduct related clinical trials, while the clinical translation of novel treatments from preclinical studies or *in vitro* drug screening should proceed with caution due to unguaranteed efficacy and safety profiles.

## Introduction

In December 2019, a series of pneumonia cases, now known as Coronavirus Disease 2019 (COVID-19), occurred in Wuhan, Hubei Province, China. A novel coronavirus was later identified as the cause of COVID-19 (1).

By February 11, 2020, a total of 72,314 cases had been reported in mainland China with 44,672 (61.8%) confirmed cases. A total of 1023 patients died, with a case fatality rate of 2.3%, and most of the deaths were in patients over 60 years of age. Among the confirmed cases, severe cases and critical cases accounted for 13.8 and 4.7%, respectively.

The Coronavirus Study Group of the International Committee on Taxonomy of Viruses assessed the novelty of the novel coronavirus and formally recognized it as a sister to severe acute respiratory syndrome coronaviruses (SARS-CoVs), designating it as severe acute respiratory syndrome coronavirus 2 (SARS-CoV-2) based on phylogeny, taxonomy and established practice (2).

However, unlike the SARS that occurred in 2003, SARS-CoV-2 infection not only leads to pneumonia and acute respiratory distress syndrome (ARDS) but is also associated with significant multiple organ dysfunction syndrome (MODS). The name of COVID-19 was chosen recently by the World Health Organization to cover the diverse clinical manifestations and reflect the complexity of the disease. Common complications among COVID-19 patients include shock, ARDS, arrhythmia, and acute cardiac injury (1). Especially for patients who require ICU care, significant cardiovascular impairment has already been observed, characterized by elevation of cardiac biomarkers, abnormalities in electrocardiography and echocardiography, and eventual circulatory failure. Cardiovascular impairment is now drawing growing attention in clinical practice, and the American College of Cardiology has already released a clinical bulletin on Feb 13, 2020, to address the cardiac implications of COVID-19 (3).

Inflammation plays an important role in the development of cardiovascular impairment and even MODS. As a cardiologist and a member of the high-level expert group appointed by the National Health Commission to fight COVID-19, during the clinical practice, I found that the experiences in cardiovascular anti-inflammatory therapy might be instructive in the management of COVID-19, especially those severe cases. Therefore, in this article, we would like to summarize the related available information and share our perspectives.

## Mechanism of Cardiovascular Impairment in COVID-19

The main reasons for cardiovascular impairment in COVID-19 patients can be summarized as follows.

### Direct Infection

**Figure 1:**
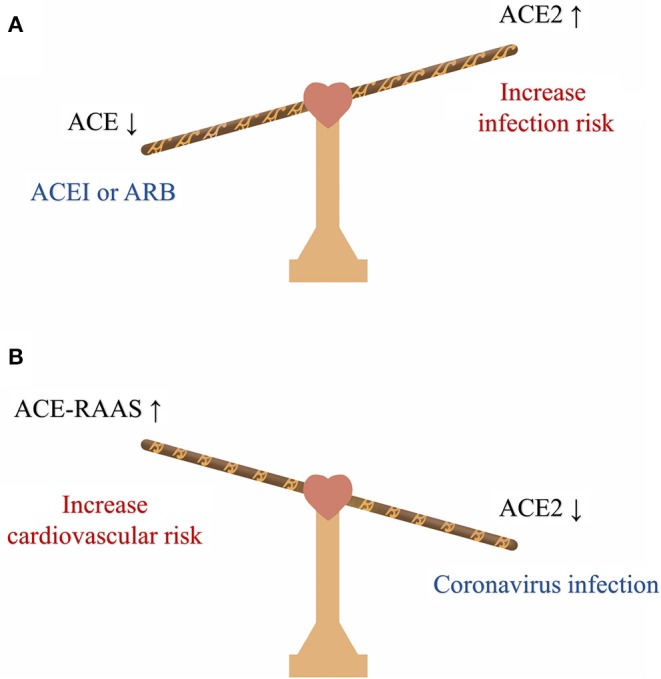
The balance between ACE and ACE2 in COVID-19. **(A)** Treatment with ACEI or ARB increases the expression of cardiac ACE2 and could further increase the risk of coronavirus infection. **(B)** Coronavirus infection can downregulate ACE2, further activate the RAAS system and increase the cardiovascular burden. ACE, angiotensin-converting enzyme; ACEI, angiotensin-converting enzyme inhibitor; ARB, angiotensin receptor blocker; RAAS, renin-angiotensin-aldosterone system.

The virus might directly infect the myocardial tissue and lead to cardiac injury. Cardiac injury has been noted as a protruding clinical feature in COVID-19 patients. In a study of 138 patients, 10 patients were diagnosed with cardiac injury and 8 of them required ICU care, accounting for 22% of all the severe cases. Compared with the non-ICU patients, ICU patients had higher level of hypersensitive troponin I and creatine kinase–MB, indicating that cardiac injury is associated with the disease severity (1). SARS-CoV-2 and SARS-CoV share the same functional host-cell receptor, angiotensin-converting enzyme 2 (ACE2) for cell entry (4), but the affinity of ACE2 for SARS-CoV-2 is approximately 10- to 20-fold higher than that for SARS-CoV (5). ACE2 is highly expressed in both the lung and heart (6), and the SARS-CoV viral RNA has been detected in autopsied heart samples from SARS patients (7). However, large-scale autopsy or biopsy studies are still required to further confirm the myocardial infection in COVID-19 by the tissue viral RNA detection or *in situ* hybridization at heart and endothelium. In addition, it is worth noticing that both blockades of AT1 receptors and inhibition of Ang II synthesis would increase the expression of cardiac ACE2 (8); therefore, for patients with hypertension or congestive heart failure (HF), regular treatment with ACE inhibitors or angiotensin receptor blockers (ARB) could further increase the risk of coronavirus infection (Figure 1). However, the causal relationship between ACEI/ARB intake and increased viral load and deleterious outcomes in COVID-19 is still uncertain. Animal studies even showed a protective effect of ARB in lung injury during SARS-CoV infection (9, 10). Considering the solid evidence of the beneficial effect of ACEI/ARB in cardiovascular diseases, it is currently not recommended to discontinue the RASS inhibition treatment in COVID-19 (11).

### Cytokine Storm

Similar to SARS-CoV and MERS-CoV infection, SARS-CoV-2 infection can also induce excessive and aberrant host immune responses, leading to a cytokine storm (12). Studies have shown increased amounts of cytokines, such as IL-1β, IL-1ra, IL-6, TNF-α, IL-7, IL-8, IL-9, IL-10, FGF basic, G-CSF, GM-CSF, IFN-γ, IP-10, MCP-1, MIP-1a, MIP-1b, in the serum of COVID-19 patients, and the cytokine storm was associated with disease severity (1, 13). An autopsy study of a COVID-19 patient also revealed that there were a few interstitial mononuclear inflammatory infiltrates in the heart tissue; besides, the flow cytometric analysis of peripheral blood found that CD4 and CD8 T cells were hyperactivated and the concentration of highly proinflammatory Th17 cells significantly increased (14). Cytokines play an important role in the immune response to defend against viral infections; however, it has also been recognized that dysregulated and excessive immune responses may cause immunopathology. Inflammation after infection can be progressively amplified through positive feedback and eventually form a cytokine storm, leading to systematic self-attack, which is a well-established explanation for MODS during coronavirus infection (15, 16).

### Aggravation of Existing Cardiovascular Diseases

SARS-CoV-2 infection is more likely to affect older patients with underlying cardiovascular comorbidities (17). According to a study, 4.2% of the confirmed cases and 22.7% of deaths have cardiovascular comorbidities (18). The fatality rate of patients with comorbidities was much higher than that of patients without comorbidities, and the fatality rate of patients with cardiovascular diseases (10.5%) was the highest (18). Therefore, COVID-19 patients are at risk of acute cardiovascular events. Secondary infection, disorder of sodium and water homeostasis, hypoxia, tissue hypoperfusion, and shock occurring during COVID-19 can all result in the aggravation of existing cardiovascular diseases and trigger severe events, such as acute coronary syndromes or exacerbation of HF. Additionally, a study has demonstrated that SARS-CoV infection can lead to the downregulation of ACE2 and activate the renin-angiotensin-aldosterone system (RASS), which would further increase the cardiovascular burden and contribute to adverse outcomes (7) (Figure 1).

In addition to the three mechanisms, the treatment with non-steroidal anti-inflammatory drugs (NSAIDs), glucocorticoids, and anti-viral agents, such as lopinavir/ritonavir (LVP/r), interferon-α (IFN-α), ribavirin, and azithromycin, could further increase the cardiovascular risk of COVID-19 patients and bring additional challenges. The harmful effects of NSAIDs and glucocorticoids on the cardiovascular system have been well-demonstrated by numerous studies that they can increase the risk of all cardiovascular events, myocardial infarction (MI), HF, and cerebral infarction (19, 20). LPV/r can induce cardiac conduction alteration and QTc and/or PR interval prolongation, further leading to atrioventricular block and torsade de pointes. LPV/r may also increase the risk of MI (21). Besides, protease inhibitor therapy has been associated with hyperglycemia, hyperlipidemia, and lipodystrophy (22) and such metabolic disturbances were also verified in patients treated with LPV/r (23). IFN-α is associated with hypertension, hypertriglyceridemia, and various cardiovascular adverse reactions and has been given a US boxed warning for its potential risk of ischemic disorders (24). A statement from the American Heart Association has announced that IFN-α can cause numerous direct cardiotoxicities, including arrhythmias, MI, and cardiomyopathy, and can also exacerbate underlying myocardial dysfunction (25). A US boxed warning has been issued for ribavirin that the hemolytic anemia associated with ribavirin may worsen underlying cardiac disease and lead to fatal and non-fatal MI (26). A recent study has shown that azithromycin could reinforce the anti-viral effect of hydroxychloroquine (27); however, its proven risk of severe QT prolongation should also be considered (28), especially when it is combined with hydroxychloroquine to treat the elderly COVID-19 patients (29).

## Rationale for Cardiovascular Anti-Inflammatory Therapy in COVID-19

Cardiovascular protective strategies are urgent for the prevention and management of severe adverse cardiovascular events, which is of great significance to the overall prognosis of COVID-19 patients. The clinical bulletin released by the American College of Cardiology has issued several points of clinical guidance regarding cardiac complications (3), and the Chinese Society of Cardiology of Chinese Medical Association also developed an expert consensus on the clinical management of patients with emergent high-risk cardiovascular disease during the epidemic period (30). However, whether the conventional treatment is sufficient to overcome such challenges and whether any additional strategy to further reduce the risk of cardiovascular attack is needed in severe cases of COVID-19 remain unclear.

Excessive inflammation should be considered as a promising target because it plays an important role in all three mechanisms described above. It has already been demonstrated that for myocarditis with or without viral trigger, inflammation is implicated in the development of both acute cardiac injury and subsequent dilated cardiomyopathy (31). There is also abundant evidence that inflammation participates in various cardiovascular diseases, such as coronary artery disease (CAD) and HF. Especially in atherosclerosis, inflammation promotes the formation, destabilization, and rupture of atheromatous plaques and has already been recognized as an independent risk factor and prognostic predictor (32).

Therefore, conventional cardiovascular treatment plus anti-inflammatory therapy is a reasonable enhanced strategy for better management of cardiovascular impairment in severe cases of COVID-19. In addition, as the inflammatory attack on different organs shares numerous similar mechanisms and pathways, such as the inflammatory response under ischemia/reperfusion injury (IRI) of the heart, liver, and kidney (33), suppression of the systematic inflammatory response will not only exert cardiovascular protection effect but also have potential benefits for MODS. Rheumatologists have also focused on the dysregulated inflammation and suggested that there might be a “window of opportunity” for immunosuppressive strategies used to treat rheumatic diseases to serve as strong allies in the fight against COVID-19 (34, 35).

## Verified Cardiovascular Anti-Inflammatory Therapies

Many clinical trials have been conducted in the past decade to directly test the feasibility of using different anti-inflammatory agents for cardiovascular protection under various conditions, mainly including CAD, myocardial IRI, HF, myocarditis/dilated cardiomyopathy, and rheumatic diseases [rheumatoid arthritis (RA), psoriatic arthritis, etc.]. Accumulating evidence has supported the efficacy of this novel strategy in improving cardiovascular outcomes. Here, we reviewed the currently available cardiovascular anti-inflammatory therapies that have been verified in clinical trials with positive results (36–47). The detailed information of these trials is listed in Table 1.

**Table 1:** Clinical trials of anti-inflammatory therapy for cardiovascular protection with positive outcomes.

**Clinical trial**	**Treatment**	**Target and mechanism**	**Patients**	**Outcomes**	**Reference**
**Cardiovascular protection in rheumatoid arthritis**					
NCT01566201	Anakinra	IL-1R	Rheumatoid arthritis	Improvement in endothelial, coronary aortic function and left ventricular myocardial deformation and twisting after CAD	(33)
ENTRACTE	Etanercept	TNF-α	Rheumatoid arthritis	Rare cardiovascular events in both groups.	Preliminary results (34)
	Tocilizumab	IL-6			
**Coronary artery disease**					
CANTOS	Canakinumab	IL-1β	Previous MI and hsCRP≥2 mg	Lower rate of nonfatal MI, nonfatal stroke, cardiovascular death, and hospitalized UA leading to urgent revascularization	(35)
LoDoCo	Colchicine	Central pathway^*^	SCAD	Prevention of ACS, out-of-hospital cardiac arrest, and non-cardioembolic ischemic stroke	(36)
COLCOT	Colchicine	Central pathway^*^	Within 30 days after a MI	Lower risk of cardiovascular death, resuscitated cardiac arrest, MI, stroke, and hospitalized UA leading to urgent revascularization)	(37)
**Myocardial ischemia/reperfusion injury**					
NCT01491074	Tocilizumab	IL-6	NSTEMI	Attenuated hsCRP and primarily PCI-related hsTnT release	(38)
**Heart failure**					
CANTOS	Canakinumab	IL-1β	Previous MI and hsCRP≥2 mg	Dose-dependent reduction in HHF and the composite of HHF or HF–related mortality	(39)
ACCLAIM	Immunomodulation therapy	Macrophages	NYHA II-IV chronic HF	Reduction in all-cause mortality and cardiovascular admission in patients with no history of MI or with NYHA II HF,	(40)
STAR-heart	Intracoronary bone marrow cell therapy	Resident cardiac macrophages^#^	Chronic HF due to ischemic cardiomyopathy	Improvement in ventricular performance, quality of life and survival	(41)
ixCELL-DCM	Ixmyelocel-T^§^	Bone marrow mononuclear cells^¶^	NYHA III or IV symptomatic HF due to ischemic dilated cardiomyopathy	Improvement in all-cause mortality, cardiac admissions, HF admissions, and left ventricular function	(42)
**Chronic myocarditis/dilated cardiomyopathy**					
CZECH-ICIT	Steroids and azathioprine	T cells suppression	Dilated cardiomyopathy and increased HLA expression on biopsy specimens	Long-term benefit in LVEF, LVV, LVDd, and NYHA class	(43)
TIMIC	Steroids and azathioprine	T cells suppression	Virus-negative myocarditis with chronic HF	Improvement in LVEF, LVV, LVD, and NYHA class	(44)

* *Central pathway refers to the immune pathway linking IL-1β, TNF-α, and IL-6*.

*Patients' own blood was stressed to induced cell death, and then the mixture of apoptotic cells was injected intramuscularly into the same patient*.

*Macrophages that phagocytose apoptotic cells downregulate proinflammatory cytokines and upregulate anti-inflammatory cytokines*.

# *Stem cells were taken up by resident cardiac macrophages which would exert cardioprotective effects*.

§ *Intramyocardial injection of expanded bone marrow–derived mesenchymal stem cells with macrophages activated ex vivo*.

¶ *Bone marrow mononuclear cells express the anti-inflammatory cytokine IL-10 to exert protective role by limiting T-cell recruitment*.

*IL-1R, interleukin-1 receptor; CAD, coronary artery disease; TNF-α, tumor necrosis factor-α; IL-6, interleukin-6; IL-1β, interleukin-1β; MI, myocardial infarction; hsCRP, high-sensitivity C-reactive protein; UA, unstable angina; SCAD, stable coronary artery disease; ACS, acute coronary syndrome; NSTEMI, non-ST segment elevation myocardial infarction; PCI, percutaneous coronary intervention; hsTnT, high-sensitivity troponin T; HHF, hospitalization for heart failure; HF, heart failure; NYHA, New York Heart Association; IL-10, interleukin-10; HLA, human leukocyte antigen; LVEF, left ventricular ejection fraction; LVV, left ventricular volume; LVDd, left ventricular diastolic dimension*.

## Promising Prospects

Based on the above review and summarization, there are several perspectives that we can conclude to possibly guide the selection of anti-inflammatory therapies in COVID-19.

### Targeting the Central Pathway (IL-1β, TNF-α, IL-6)

The immune pathway linking IL-1β, TNF-α, and IL-6, known as the central pathway, has long been implicated in atherosclerosis and is considered to play an important role in CAD (48). The significant effect of such a central pathway has also been recognized in many other fields of cardiovascular research (49–52), and a large portion of the clinical trials reviewed above was designed to target this pathway. Activation of the central pathway has already been observed in COVID-19 and should, therefore, be considered as a promising target (13). A multicenter, randomized controlled trial (ChiCTR2000029765) has been registered to evaluate the efficacy and safety of IL-6 blockade using tocilizumab in COVID-19. According to the preliminary treatment results currently released, among the 14 patients recruited (maximum age 82), including 9 severe cases and 2 critical cases, tocilizumab significantly improved the fever symptom and lung function and also accelerated the absorption of lung lesions. In addition to the benefits reviewed above, it is worth mentioning that tocilizumab also has a potential electrophysiological protective effect. Increased IL-6 level has been associated with acquired long QT-syndrome in patients with systemic inflammation, leading to higher risks for arrhythmias such as torsade de pointes (53). In RA patients, tocilizumab treatment led to a rapid and significant QT shortening correlating with the decrease in CRP and cytokine levels, which might benefit the overall mortality (54, 55). Such anti-arrhythmic potential may further support the application of tocilizumab in COVID-19 patients to counteract the risk of adverse QT prolongation and related life-threatening arrhythmias associated with elevated IL-6 and the anti-viral agents. A series of clinical trials on chloroquine in the treatment of COVID-19 are also currently underway and have revealed considerable benefits (27). Chloroquine has now been included in the 6th version of the Diagnosis and Treatment Plan for Novel Coronavirus Pneumonia (56). In addition to its direct antiviral effect (57), chloroquine might also exert anti-inflammatory effects by inhibiting the central pathway (58). Hydroxychloroquine (HCQ) has long been used to reduce inflammation in patients with RA and lupus. In a nationwide cohort study, HCQ use was associated with decreased CAD risk in the RA population (59). Besides, an OXI trial (NCT02648464) is currently underway to study the effect of HCQ on the prevention of recurrent cardiovascular events among MI patients (60). Therefore, we recommend targeting the central pathway by blockade of IL-6 (tocilizumab), IL-1β [canakinumab (IL-1β monoclonal antibody), anakinra (IL-1 receptor antagonist)], and TNF-α (etanercept, infliximab) to control the cytokine storm in the acute phase of COVID-19, thereby reducing cardiovascular impairment and even mitigating MODS.

### Balancing the T-helper-1 (Th1) Cell and T-helper-2 (Th2) Cell Response and Promoting the Secretion of Anti-Inflammatory Cytokines

Studies of IRI and myocarditis have revealed that the Th1 response, characterized by the expression of multiple proinflammatory cytokines, is activated in the early disease phase and is associated with acute cardiac injury (61–63), while the Th2 response dominates later, promoting the resolution of acute inflammation and tissue repair. M2 macrophage polarization was found to be a significant change contributing to the transition from the Th1 to Th2 response, and monocyte-derived IL-10 is a well-recognized Th2-related anti-inflammatory cytokine that is highly expressed in the reparative phase and inhibits the Th1 response (64, 65). Early activation of the Th2 response or increased IL-10 expression in IRI and myocarditis could significantly inhibit the secretion of Th1-related proinflammatory cytokines and reduce myocardial necrosis (66–69). Patients with COVID-19 had high amounts of IL-1β, IFN-γ, IP-10, and MCP-1, indicating an activated Th1 response; besides, SARS-CoV-2 could also initiate increased secretion of Th2 cytokines, especially IL-10, which is different from SARS-CoV infection (70). Therefore, the implantation of mesenchymal stromal cells (MSCs) from allogeneic donors with an activated Th2 response or *ex vivo* bone marrow-derived MSCs after M2 macrophage polarization might increase the level of IL-10 in the acute phase of COVID-19 and serve as a possible solution to inflammation-mediated damage. In addition to the application in cardiovascular diseases reviewed above, cellular therapy using MSCs has also shown efficacy in the management of ARDS and is now being evaluated in phase 1/2 trials (12).

### Long-Term Anti-Inflammatory Therapy in the Recovery Phase

Inflammation in the acute phase can lead to extensive injury; however, it should also be noted that after the acute damage, chronic residual inflammation that occurs with fibrosis during the reparatory phase can also result in persistent organ dysfunction. Studies of HF due to ischemic cardiomyopathy and chronic myocarditis have found that chronic residual inflammation is associated with myocardial fibrosis and adverse ventricular remodeling (49, 71–73). In addition, the long-term inflammatory status is also a hazard to atherosclerosis (32). During the follow-up of SARS and MERS patients, it was also observed that in those who survive intensive care, residual immune responses could lead to long-term lung damage and fibrosis, causing functional disability and reduced quality of life (74, 75). Therefore, in addition to the control of acute injury, it is also of great significance to conduct long-term follow-up after admission to monitor residual inflammation in COVID-19 patients, especially those with severe clinical manifestations or intense acute inflammatory responses. After excluding potential contraindications, long-term anti-inflammatory therapy, such as steroids, azathioprine, and canakinumab, should be considered to reduce the residual inflammation and prevent further chronic structural and functional damage.

## Discussion

**Figure 2:**
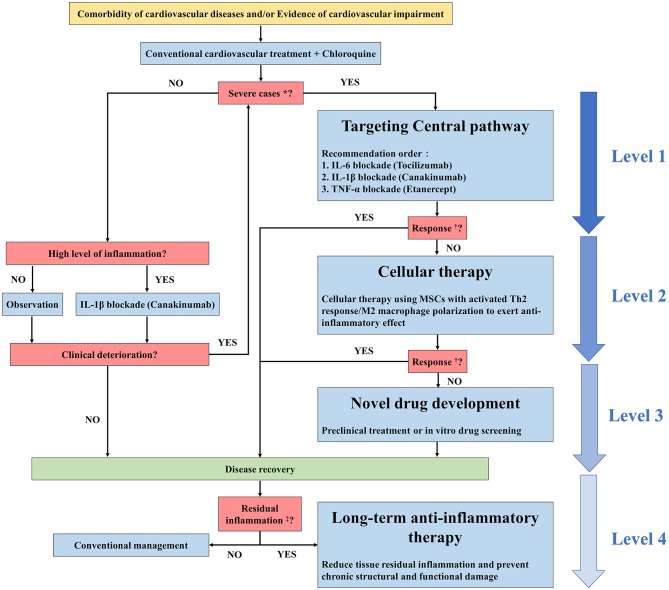
Possible algorithm regarding the selection of anti-inflammatory therapy for COVID-19 patients with cardiovascular impairment. *Severe cases refer to patients with the following conditions according to the 6th version of the Diagnosis and Treatment Plan for Novel Coronavirus Pneumonia: respiratory rate≥30 bpm; SpO2 ≤ 30% at rest; PaO2/FiO2 ≤ 300 mmHg; progression on chest images>50% within 24 to 48 h; respiratory failure that requires mechanical ventilation; shock; organ dysfunction that requires ICU admission. ^†^Response refers to the response to anti-inflammatory therapy. The criteria include but are not limited to decreased white blood cell counts, decreased hsCRP levels, decreased cytokine levels, significant symptom improvement, and significant improvement in chest images. Residual inflammation refers to evidence of a persistent state of high inflammation characterized by high levels of hsCRP and/or serum cytokines.

Hyperactivated inflammation is fundamental in the development of cardiovascular impairment and even MODS in COVID-19. In addition to conventional cardiovascular treatment, anti-inflammatory therapy is a reasonable strategy to further enhance cardiovascular protection and potentially mitigate MODS. By reviewing the inflammatory features and current promising treatments of COVID-19 as well as cardiovascular anti-inflammatory therapies that have been verified in clinical trials with positive results, we believed that targeting the central pathway (IL-1β, TNF-α, IL-6), balancing the Th1 and Th2 response, and administering long-term anti-inflammatory therapy should be considered as promising strategies to control cardiovascular impairment or even MODS during the acute and recovery phases of COVID-19. The experiences in cardiovascular anti-inflammatory therapies might be of great value to the management of COVID-19 patients and we recommended to take such experiences as references for clinical practice and conduct related clinical trials. We here propose a possible algorithm regarding the selection of anti-inflammatory therapy for COVID-19 patients with or at high risk of cardiovascular impairment (Figure 2).

Despite all the beneficial effects described above, it is also important to pay attention to the potential adverse cardiovascular effects of these drugs. For tocilizumab, hypercholesterolemia and hypertension are both common adverse events, prompting concern about increased cardiovascular risk (76). Especially, tocilizumab was widely noted to induce a proatherogenic lipid profile with increased serum levels of low-density lipoprotein cholesterol and total cholesterol (77–79). However, these changes can be improved by concomitant therapy with statins (80). For TNF inhibitor (TNFi), although not universally acknowledged, it might not be beneficial to HF. Severe HF remains a contraindication to TNFi treatment in RA patients. A clinical trial showed that high-dose infliximab could be harmful to patients with moderate-to-severe HF (81). For RA populations, a cohort study found that TNFi might increase the risk of both first hospitalization and exacerbation of HF in elderly patients with RA (82). Additionally, for hydroxychloroquine, a recent study pointed out that hydroxychloroquine could lead to unwanted QT interval prolongation by blocking the KCNH2-encoded hERG/Kv11.1 potassium channel, thereby increasing the risk of drug-induced torsade de pointes and sudden cardiac death (83). Therefore, biochemical indicators, hemodynamic parameters, and cardiac electrophysiology profiles should be monitored in clinical practice to avoid drug-induced adverse effects on cardiovascular risk factors, cardiac function, or lethal arrhythmias. Besides, corresponding treatment, such as lipid-lowering or antihypertensive medications should be prescribed if necessary.

Currently, a large number of novel therapies from preclinical studies or *in vitro* drug screening have been registered and accelerated into clinical practice. However, the safety profiles of these therapies have always not been well-characterized, especially for elderly COVID-19 patients with hepatic or renal dysfunction. In addition, as many of the therapies were proven to be effective only by *in vitro* experiments or poorly designed small-scale clinical studies, the exact benefits were also not guaranteed. Therefore, clinical translation of novel treatments from preclinical studies or *in vitro* drug screening should proceed with caution due to unguaranteed efficacy and safety profiles. Recently, experts from multiple research institutions in China raised their criticism and announced an urgent call for increasing the scientific rigorousness of clinical trials on COVID-19 (84). Considering timeliness and safety, we suggest prioritizing cardiovascular protective strategies that have been proven by large-scale clinical trials for proof-of-concept studies and clinical application on COVID-19 instead of rushing into new drug research and development.

## Data Availability Statement

The original contributions presented in the study are included in the article/supplementary materials, further inquiries can be directed to the corresponding author/s.

## Author Contributions

LW contributes to the literature search, manuscript preparation, and manuscript editing. YZ contributes to concept, design, manuscript preparation, and manuscript editing. SZ contributes to concept, design, definition of intellectual content, and manuscript review.

## Conflict of Interest

The authors declare that the research was conducted in the absence of any commercial or financial relationships that could be construed as a potential conflict of interest.
